# Risk Factors of Stroke in Western and Asian Countries: A Systematic Review and Meta-analysis of Prospective Cohort Studies

**DOI:** 10.1186/1471-2458-14-776

**Published:** 2014-07-31

**Authors:** Xuetao Chen, Liang Zhou, Yanqi Zhang, Dali Yi, Ling Liu, Wen Rao, Yazhou Wu, Dihui Ma, Xiaoyu Liu, Xiao-Hua Andrew Zhou, Hui Lin, Dixiang Cheng, Dong Yi

**Affiliations:** Department of Health Statistics, College of Preventive Medicine, Third Military Medical University, PO Box 400038, Chongqing, China; Department of Information, Xinqiao Hospital, Third Military Medical University, Xinqiao Street, Chongqing, 400037 China; Department of Epidemiology, College of Preventive Medicine, Third Military Medical University, Chongqing, 400038 China; Department of Biostatistics, School of Public Health and Community Medicine, University of Washington, Seattle, 98124-6108 USA; Department of Epidemiology, Institute of tropical medicine, Third Military Medical University, Chongqing, 400038 China; School of Software Engineering, Chongqing University of Posts and Telecommunications, Chongqing, 400065 China

**Keywords:** Stroke, Risk factors, Prospective study, Epidemiology, Systematic review, Public health

## Abstract

**Background:**

There has been an increasing trend in the incidence of stroke worldwide in recent years, and the number of studies focusing on the risk factors for stroke has also increased every year. To comprehensively evaluate the risk factors of stroke identified in prospective Western and Asian cohort studies.

**Methods:**

Population-based cohort studies on stroke were searched in databases (PubMed, EMBASE, Web of Science, Google Scholar, etc.), and the library of the Third Military Medical University was manually searched for relevant information. A meta-analysis of Western and Asian studies on risk factors was performed. The pooled hazard ratios (HRs) with 95% confidence intervals (CIs) were calculated to assess the final group of cohort studies.

**Results:**

After screening, 22 prospective cohort studies were included in the analyses of this investigation. Two factors, smoking and alcohol consumption, showed statistically significant differences between Western and Asian populations, and the results were as follows (W/A): 2.05 (95% CI, 1.68 ~ 2.49)/1.27 (95% CI, 1.04 ~ 1.55) and 0.89 (95% CI, 0.76 ~ 1.04)/1.28 (95% CI, 1.07 ~ 1.53). The factor BMI = 18.5-21.9 kg/m^2^ showed statistically significant differences only in Western populations, 0.96 (95% CI, 0.93 ~ 0.99); the factor SBP = 120-139 mm Hg showed statistically significant differences only in Asian populations, 2.29 (95% CI, 1.04 ~ 5.09).

**Conclusions:**

The prevalences of risk factors affect the stroke morbidity in Western and Asian populations, which may be biased by race. The meta-analysis of population-based studies suggests that different preventive measures should be adopted for Western and Asian population groups that are at high risk for stroke.

**Electronic supplementary material:**

The online version of this article (doi:10.1186/1471-2458-14-776) contains supplementary material, which is available to authorized users.

## Background

Stroke is not only the second leading cause of death worldwide but also one of the main causes of adult-acquired disabilities [1, 2]. In certain countries, the incidence of stroke has increased over time. For example, the incidence of stroke in the city of Frederiksberg in Denmark increased from 618/100,000 during the period from 1972–1974 to 1,190 per 100,000 during the period from 1989–1990 [3], and the incidence of stroke among males in Gothenburg, Sweden increased by 35% per year from 1985 to 1990 [4]. In recent years, the incidence of stroke has gradually increased among younger populations. In a region of Western Norway, the incidence of ischemic stroke among young adults was 11.4/100,000 between 1988 and 1997, with women accounting for the majority of patients who experienced a stroke before reaching 30 years of age and males accounting for the majority of patients who experienced a stroke after 30 years of age [5]. In India, the prevalence of stroke is 1.54, with a death rate of 0.6 per 1,000 population; the age-adjusted stroke mortality is believed to be 60% higher in South-East Asia than in European populations [6]. The same situation exists in Iran; a systematic review reported that the stroke prevalence for various ages in Iran ranges from 23 to 103 per 100,000 populations [7].

There have been numerous recent research reports regarding the risk factors for stroke, which include not only unhealthy lifestyle habits such as smoking and drinking but also hypertension, diabetes, and a family history of diseases, among other traits [8–18]. However, different sets of risk factors and risk intensities have been identified by various studies, and no comprehensive systematic review has compiled and summarized the findings of these studies. In this investigation, based on a large number of prospective cohort studies from around the world, a systematic review and a meta-analysis were conducted to summarize the risk factors for stroke in Western and Asian populations.

## Methods

Our investigation was conducted as suggested by the Meta-analysis of Observational Studies in Epidemiology (MOOSE) group [19]. A detailed query strategy and inclusion and exclusion criteria were developed, and the relevant literature was screened for inclusion using these standards. Two research groups were established. The first research group was responsible for searching for and reviewing relevant studies and extracting the original data from these investigations. The second group evaluated the quality of the included studies. Finally, pooled statistics describing various effects were calculated.

### Systematic literature search

The following electronic literature databases were searched: PubMed, EMBASE (the Excerpta Medica Database), Web of Science, Google Scholar, the Chinese Biomedical Literature Database (CBM), the integrated Chinese Medical Citation Index/Chinese Medical Current Contents (CMCI/CMCC) database, and the Cochrane Central Register of Controlled Trials (CENTRAL) database of the Cochrane Library. The following search terms were utilized: ((((((((stroke) OR ischemic) OR hemorrhagic) OR observational study) OR cohort study) AND risk factors) OR incidence) OR mortality) AND prospective study. In addition, the library of the Third Military Medical University was manually searched for relevant information. The period specified in these searches ranged from the creation date of each database to May 2013.

### Inclusion and exclusion criteria

Studies were included if they met the following criteria: ① The included studies were prospective, population-based cohort studies; ② The included studies involved research participants who were volunteers from a community or hospital, with no limitations placed on sex, age, or type of stroke, although the participants were required to be free from any complicating infectious or traumatic conditions; ③ The included studies were required to provide HRs for the risk factors; ④ The observation endpoint of the included studies was the onset of stroke; ⑤ The included studies were required to base the determination of disease on internationally adopted professional diagnostic criteria[20, 21]; ⑥ Comparability of the cohorts was required on the basis of the design or analysis; ⑦ The included studies were required to provide a full contingency table and/or adjusted *HR*/*RR* (relative risk) values.

The exclusion criteria included the following: ① Studies examining populations that might be affected by interactions between genes and the environment, such as the Asian-American population, were excluded; ② Case control studies and other retrospective studies were excluded from consideration; ③ Duplicate publications, animal experiments, reviews, and systematic reviews were excluded from consideration.

### Selection and data extraction

The retrieved literature was carefully reviewed, and various information was extracted for each examined study, including the study’s first author, publication year, sample size, study type, population characteristics, study endpoint, and *HR/RR* values and 95% confidence intervals (CIs) (or a contingency table). The data were independently extracted in a blinded manner by two researchers (LZ and XC) from the first research group. A third researcher (LL) from this research group was consulted to resolve discrepancies in the extracted data, and a consensus was reached through discussion.

### Quality assessment and statistical analyses

The included observational studies were subjected to a comprehensive quality assessment using the Newcastle-Ottawa Scale as a guide [22]. This quality evaluation was performed in a blinded manner by two researchers (YW and YZ) from the second research team, who assigned quality scores to the included studies. The studies that were given different quality scores by the two researchers were referred to a third researcher (DY) from this research team for evaluation, and a final quality score was obtained.

Q tests were conducted to determine the heterogeneity of the included studies. If I^2^ ≥ 50% and P < 0.1, a random-effects model was used to combine the studies; by contrast, if I^2^ ≤ 50% and P > 0.1, a fixed-effects model was used to combine the studies. The adjusted HR was used to measure the effect sizes for the pooled prospective cohort studies, and 95% CIs were determined for the pooled studies. Egger’s regression test was used to verify the absence of publication bias, and by model transformation between the fixed effects model and random effects model for the sensitivity analysis. The fundamental approach utilized in this non-parametric method involves first trimming (removing) small sample studies that cause funnel plot asymmetries. The trimmed symmetric remainder is then used to estimate the center value of the funnel plot, and the removed studies and their corresponding estimated missing values are then entered into the plot on both sides of this center value [23], enabling an assessment of the stability of the entire systematic review. All of the statistical analyses were conducted using the Stata 11.0 software package.

## Results

### Included studies

**Figure 1:**
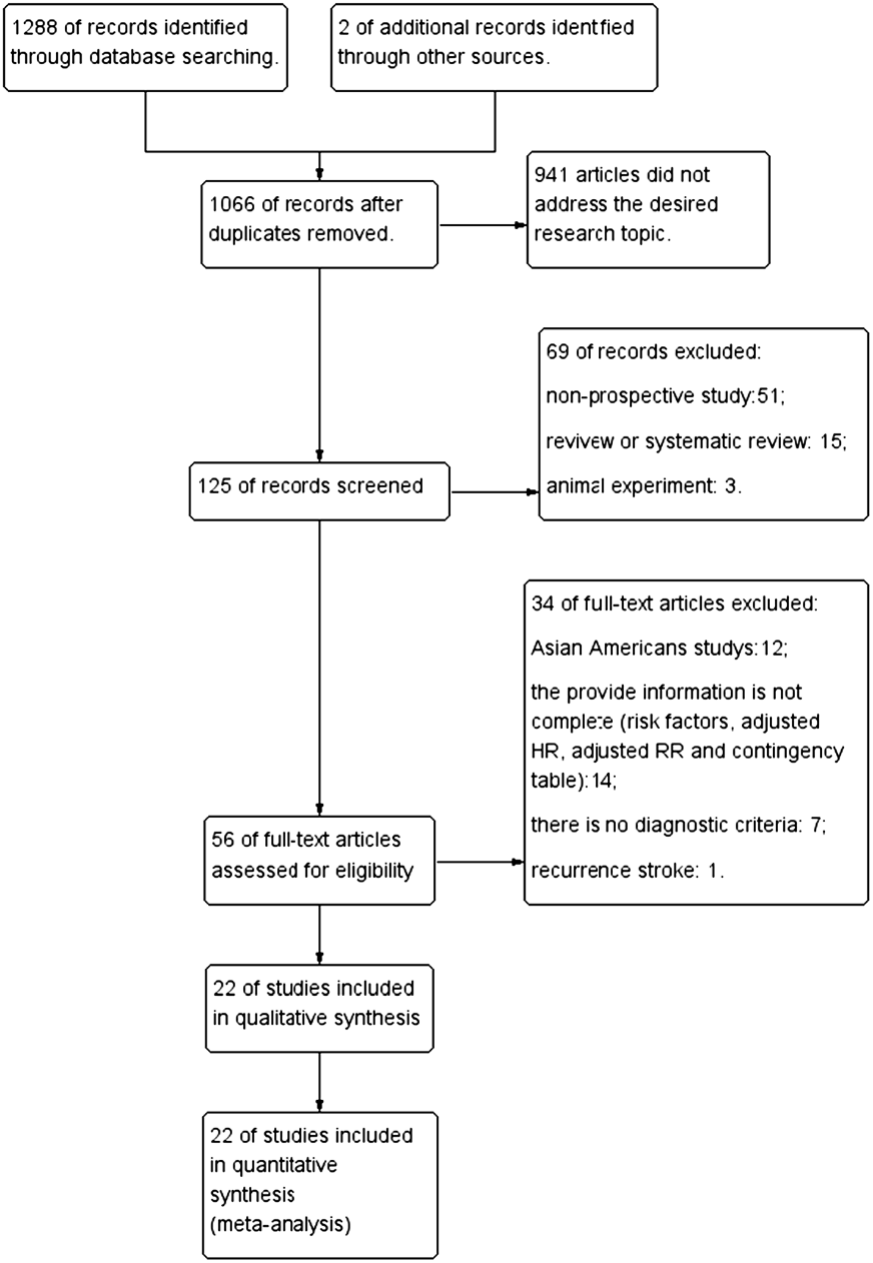
**Flow diagram.**

The database search resulted in the identification of 1,288 articles; in addition, 2 conference-related publications were identified from other sources. In total, 224 of these articles were duplicates that had been identified in multiple electronic databases, and 941 articles did not address the desired research topic; thus, 125 studies were included in the initial screening (including 123 articles and two conference-related publications). The initial screening of the abstracts of these studies resulted in the exclusion of 51 non-prospective cohort studies, 15 reviews or systematic reviews, and 2 animal studies. The full-text versions of the remaining 56 articles were then assessed, and 34 articles that did not meet the inclusion criteria were excluded from consideration. Ultimately, 22 prospective cohort studies were included in this systematic review and meta-analysis (Figure 1 and Table 1).

**Table 1:** **Characteristics of the prospective studies on stroke**

First author	Publication year	Sex	Research site	Type of stroke	Sample (person-years)	Sample who had a stroke	Risk factors (Sample who had a stroke)	Source of the cohort
A.G. Shaper [11]	1991	Men	England	Ischemic & hemorrhagic	7,735	110	Smoking (98)	The British Regional Heart Study [24]
Kathryn M. Rexrode [25]	1997	Women	England	Ischemic & hemorrhagic	866	672	BMI (672)	The Nurses' Health Study [26, 27]
K. Berger [28]	1998	Men & women	Germany	Ischemic & hemorrhagic	12,866	39	SBP (36), Hypertension (17), Diabetes (3), Smoking (19)	The PROCAM Study^**△**^ [29, 30]
Leon A. Simons [31]	1998	Men & women	Australia	Ischemic & hemorrhagic	2,805	306	BMI (306), SBP (306), Diabetes (N/A), Coronary heart disease (N/A), AF (N/A), Smoking (N/A)	The Dubbo Study [32]
K.S. Wong [33]	1999	Men & women	Asian^◆^	Ischemic & hemorrhagic	3,186	2,403	AF (265), Diabetes (634), Ischemic heart disease (431)	The hospital participants in an Asian country
P McCarron [34]	2001	Men	England	Ischemic & hemorrhagic	4,826	293	BMI (293), SBP (293), Diabetes (13), AF (9), Smoking (87)	The Caerphilly and Speedwell Collaborative Studies [35, 36]
Yao He [37]	2003	Men & women	China	Ischemic & hemorrhagic	1,268	45	BMI (45), SBP (45), Hypertension (N/A)	The cadre's sanitarium in Xi’an, China
Helen Rodgers [38]	2004	Men & women	Northern England	Ischemic & hemorrhagic	4,440	329	BMI (329), Hypertension (150), Diabetes (24), Coronary heart disease (53), AF (32), LVH (45), Alcohol (124), Smoking (84)	The population-based survey of atrial fibrillation [39]
Yun-Mi Song (a) [40]	2004	Men	Korea	Ischemic & hemorrhagic	234,863	7,444	BMI (7,444), SBP (7,444)	The participants were provided by KNHS.
Yun-Mi Song (b) [41]	2004	Men & women	Korea	Ischemic & hemorrhagic	14,057	10,716	SBP (10,716)	The participants were provided by KNHS.
Xiao-Fei Zhang [42]	2004	Men	China	Ischemic & hemorrhagic	5,092	124	BMI (124), SBP (124), Hypertension (80), Smoking (124)	The workforce of Beijing, China
Wei Wang [43]	2006	Men & women	China	Ischemic & hemorrhagic	30,378	565	SBP (565), Smoking (188), Diabetes (57)	The CMCS study^**△**^ [44]
Per Harmsen [45]	2006	Men	Sweden	Ischemic & hemorrhagic	7,457	317	BMI (317), SBP (317), Diabetes (147), AF (37), Smoking (159)	The Multifactor Primary Prevention Study [46]
Mohsen Janghorbani [47]	2007	Women	Iran	Ischemic & hemorrhagic	121,701	2,384	Diabetes (92)	The Nurses’ Health Study [26, 27]
Truong-Minh Pham [48]	2007	Men & women	Japan	Ischemic & hemorrhagic	9,651	226	BMI (226), Hypertension (80), Smoking (108), Alcohol (61)	The participants of the Miyako Study [49, 50]
Cornelia Weikert [51]	2007	Men & women	Germany	Ischemic	25,538	85	Hypertension (74), Diabetes (14), Alcohol (27)	The European Prospective Investigation into Cancer and Nutrition [52]
Yan Wang [53]	2007	Men & women	China	Ischemic & hemorrhagic	56,510	78	Diabetes (33), Alcohol (24), Smoking (38)	Community residents of Shanghai, China
Michiaki Kubo [54]	2008	Men & women	Japan	Ischemic	6390	430	Hypertension (N/A), Diabetes (N/A), Smoking (N/A), Alcohol (N/A)	The Hisayama Study [55]
Ying Zhang [56]	2008	Men & women	American	Ischemic & hemorrhagic	4549	306	SBP (306), Hypertension (170), Diabetes (211), Alcohol (153), Smoking (221)	The Strong Heart Study [57–59]
George Howard [60]	2011	Men & women	American	Ischemic & hemorrhagic	1842	427	SBP (427), Diabetes (110), Coronary heart disease (131), AF (64), LVH (26), Smoking (89)	The REGARDS study [61]^**△**^
I Saito [62]	2011	Men & women	Japan	Ischemic & hemorrhagic	71762	2,119	BMI (2119)	The Japan Public Health Center-based prospective (JPHC) Study
Ulla Brasch Mogensen [63]	2012	Men & women	Denmark	Ischemic & hemorrhagic	240,000	988	Hypertension (304), Diabetes (145), AF (161), Smoking (362), Alcohol (260)	The COST cohort [64, 65]^**△**^

CT: Computed tomography; MRI: Magnetic Resonance Imaging; N/A, no data available; ◆China, India, Indonesia, Korea, Malaysia, the Philippines, Taiwan, Thailand, Singapore, and Vietnam; △PROCAM the Prospective Cardiovascular Muenster Study; CMCS the Chinese Muhi Provincial Cohort Study; REGARDS, the Reasons for Geographic and Racial Differences in Stroke; COST, the Copenhagen Stroke Study. KNHS, Korean National Health System.; BMI, Body mass index; SBP, Systolic blood pressure; AF, Atrial fibrillation; LVH, Left ventricular hypertrophy.

### Study characteristics

This study examined data reported by researchers from Western (North America, Europe, and Oceania) and Asian (East Asia, South Asia, and Southeast Asia) countries. In the 22 prospective cohort studies that were included in this investigation, the sample sizes ranged from a minimum of 39 cases to a maximum of 7,444 cases; 30,406 cases were examined in these studies. Eleven of the included studies involved Western populations, and the remaining 11 studies involved Asian populations. In 20 of the included studies, the outcomes included both ischemic stroke and hemorrhagic stroke, whereas the remaining 2 studies only examined ischemic stroke (Table 1).

### Synthesis of results

This study examined 7 risk factors for stroke in human populations, including biochemical indices, lifestyle choices, and medical history. A meta-analysis produced pooled HRs with 95% CI values for each of these factors.

The combined results indicated that 6 factors affected the incidence of stroke in Western populations. These factors included BMI, SBP, hypertension, diabetes, cardiac causes, and smoking. The following pooled HR values with 95% CIs were obtained for these factors: 0.96 (0.93 ~ 0.99) for a BMI of 18.5-21.9 kg/m^2^; 2.39 (1.44 ~ 3.97) for an SBP ≥140 mm Hg; 1.79 (1.39-2.30) for hypertension; 1.85 (1.43 ~ 2.38) for diabetes; 1.74 (1.47 ~ 2.04) for cardiac causes; and 2.05 (1.68 ~ 2.49) for smoking; However, 0.89 (0.76 ~ 1.04) for alcohol consumption (Table 2 and Additional files 1, 2, 3, 4, 5, 6, 7, 8).

**Table 2:** **Meta-analysis outcomes**

Risk factors	Number of references (Western)	Endpoints/Individuals (Western)	Pooled HR (Western)	Pooled 95% CI (Western)	P (Western)	Number of references (Asian)	Endpoints/Individuals (Asian)	Pooled HR (Asian)	Pooled 95% CI (Asian)	P (Asian)
**BMI (kg/m** ^**2**^ **)**										
22.0-24.9			1.000	Ref. Group				1.000	Ref. Group	
18. 5–21. 9	3^F^	447^**^/1,917	0.96^(1)^	0.93 ~ 0.99^(1)^	0.010^*^	5^R^	1,124^**^/9,985	0.97^(9)^	0.88 ~ 1.08^(9)^	0.626
≥25.0	4^R^	389^**^/1,917	1.21^(1)^	0.99 ~ 1.48^(1)^	0.159	4^R^	2,233^**^/9,985	1.28^(9)^	1.15 ~ 1.46^(9)^	0.000^*^
**SBP (mm Hg)**										
<120			1.000	Ref. Group				1.000	Ref. Group	
120-139	2^F^	14^**^/332	1.28^(2)^	0.96 ~ 1.70^(2)^	0.094	2^R^	81^**^/689	2.29^(10)^	1.04 ~ 5.09^(10)^	0.041^*^
≥140	2^R^	22^**^/332	2.39^(2)^	1.44 ~ 3.97^(2)^	0.000^*^	2^R^	30^**^/689	6.22^(10)^	2.27 ~ 17.08^(10)^	0.000^*^
**Hypertension**										
No			1.000	Ref. Group				1.000	Ref. Group	
Yes	5^R^	715/1,747	1.79^(3)^	1.39 ~ 2.30^(3)^	0.000^*^	4^R^	80^**^/825	2.84^(11)^	2.10 ~ 3.82^(11)^	0.000^*^
**Diabetes**										
No			1.000	Ref. Group				1.000	Ref. Group	
Yes	9^R^	667^**^/2,941	1.85^(4)^	1.43 ~ 2.38^(4)^	0.000^*^	5^R^	816^**^/5,860	1.95^(12)^	1.42 ~ 2.67^(12)^	0.000^*^
**Cardiac causes** ^#^										
No			1.000	Ref. Group				1.000	Ref. Group	
Yes	6^R^	558^**^/2,511	1.74^(5)^	1.47 ~ 2.04^(5)^	0.000^*^	1^F^	431/2,403	1.87^(13)^	1.45 ~ 2.42^(13)^	0.000^*^
**Cardiac causes** (Atrial fibrillation)										
No			1.000	Ref. Group			1.000	Ref. Group		
Yes	3^F^	96^**^/1,062	1.59^(6)^	1.33 ~ 1.90^(6)^	0.000^*^	1^F^	265/2,403	2.30^(14)^	1.50 ~ 3.53^(14)^	0.000^*^
**Smoking**										
No			1.000	Ref. Group				1.000	Ref. Group	
Yes^△^	10^R^	960^**^/3,115	2.05^(7)^	1.68 ~ 2.49^(7)^	0.000^*^	5^R^	350^**^/858	1.27^(15)^	1.04 ~ 1.55^(15)^	0.020^*^
**Smoking** (former)	4^R^	135^**^/3,115	1.22^(7)^	0.94 ~ 1.59^(7)^	0.135	N/A	N/A	N/A	N/A	N/A
**Smoking** (Current)	5^R^	274^**^/3,115	2.27^(7)^	1.76 ~ 2.93^(7)^	0.000^*^	N/A	N/A	N/A	N/A	N/A
**Smoking** (≤20 Cigarettes/d)	3^F^	42^**^/3,115	2.31^(7)^	1.80 ~ 2.96^(7)^	0.000^*^	N/A	N/A	N/A	N/A	N/A
**Smoking** (>20 Cigarettes/d)	3^F^	30^**^/3,115	2.99^(7)^	2.31 ~ 3.86^(7)^	0.000^*^	N/A	N/A	N/A	N/A	N/A
**Alcohol**										
No			1.000	Ref. Group				1.000	Ref. Group	
Yes^△^	4^F^	537^**^/1,708	0.89^(8)^	0.76 ~ 1.04^(8)^	0.477	3^F^	85^**^/734	1.28^(16)^	1.07 ~ 1.53^(16)^	0.011^*^

*P values for comparison between the subjects who had a stroke and those who did not; **Insufficient data ^#^Cardiac causes: Coronary heart disease, Left atrial hypertrophy, Angina, Atrial fibrillation; F: Fixed effects model, R: Random effects model; N/A, no data available. BMI, Body mass index; SBP, Systolic blood pressure; Smoking/former: smoking cessation; Alcohol: Often drunk, △The statistical significance between Western and Asian populations; (1)-(16): (Additional files 1, 2, 3, 4, 5, 6, 7, 8, 9, 10, 11, 12, 13, 14, 15, 16).

In addition, in a subgroup analysis of smoking and cardiac causes, there was statistical significance in the 4 subgroups, including atrial fibrillation, current smoking, smoking ≤20 cigarettes/d and smoking >20 cigarettes/d. The pooled HRs with 95% CI values were as follows: 1.59 (1.33 ~ 1.90), 2.27 (1.76 ~ 2.93), 2.31 (1.80 ~ 2.96) and 2.99 (2.31 ~ 3.86) (Table 2 and Additional files 5, 7).

The included studies of Asian populations indicated that 7 factors affected the incidence of stroke. These factors included BMI, SBP, hypertension, diabetes, cardiac causes, smoking and alcohol. The following pooled HR values with 95% CIs were obtained for these factors: 1.28 (1.15 ~ 1.46) for a BMI of ≥25.0 kg/m^2^; 2.29 (1.04 ~ 5.09) for an SBP of 120–139 mm Hg; 6.22 (2.27 ~ 17.08) for an SBP ≥140 mm Hg; 2.84 (2.10-3.82) for hypertension; 1.95 (1.42 ~ 2.67) for diabetes; 1.87 (1.45 ~ 2.42) for cardiac causes; 1.27 (1.04 ~ 1.55) for smoking; and 1.28 (1.07 ~ 1.53) for alcohol consumption (Table 2 and Additional files 9, 10, 11, 12, 13, 14, 15, 16).

In the subgroup analysis of cardiac causes, there was statistical significance for atrial fibrillation. The pooled HR with the 95% CI was 2.30 (1.50 ~ 3.53) (Table 2 and Additional file 12).

### Quality of the included studies

The Newcastle-Ottawa scale results revealed that 95.45% of the included studies earned above 2 stars for the NOS selection item, 77.27% of the included studies earned above 1 star for the NOS comparability item, and 36.36% of the included studies earned above 2 stars for the NOS exposure item (Table 3).

**Table 3:** **The results of the Newcastle-Ottawa scale (NOS)**

First author	Publication year	Selection	Comparability	Exposure
Chinese population				
Yao He^37^	2003	★★★	★	★★
Xiao-Fei Zhang ^42^	2004	★★★	★★	★★★
Yan Wang^53^	2007	★★★	★	★
Wei Wang ^43^	2006	★★	★	★★
Other population				
A.G.Shaper^11^	1991	★★★	★★	★★
Kathryn M. Rexrode^25^	1997	★★★	★★	★★
K. Berger^28^	1998	★★★	★★	★★★
Leon A. Simons ^31^	1998	★★★	★★	★★
P McCarron^34^	2001	★★★★	★★	★★★
Cornelia Weikert^51^	2007	★★★★	★★	★★★
Per Harmsen^45^	2006	★★★	★★	★★
Ulla Brasch Mogensen^63^	2012	★★★	★	★★
Helen Rodgers^38^	2004	★★★★	★★	★★★
George Howard^60^	2011	★★★	★★	★★
K.S. Wong^33^	1999	★★★★	★★	★★
Yun-Mi Song (a)^40^	2004	★★★★	★	★★★
Yun-Mi Song (b)^41^	2004	★★★★	★	★★
Mohsen Janghorbani^47^	2007	★★★★	★★	★★★
Truong-Minh Pham^48^	2007	★★★	★★	★★
Michiaki Kubo^54^	2008	★★★	★★	★★★
Ying Zhang^56^	2008	★★★★	★★	★★
Ulla Brasch Mogensen^63^	2012	★★★	★	★★

Note [22]: Selection: 1. Representativeness of the exposed cohort. 2. Selection of the non-exposed cohort. 3. Ascertainment of exposure. 4. Demonstration that the outcome of interest was not present at the beginning of the study. (High quality, >2 stars); Comparability: 1. Comparability of the cohorts on the basis of the design or analysis. (High quality, >1 star); Exposure: 1. Assessment of the outcome. 2. Was the follow-up long enough for outcomes to occur? 3. Adequacy of the follow-up of the cohorts. (High quality, >2 stars).

Egger's test was performed to assess the influential factors for each of the two populations that were examined (Table 4). Publication bias affected 2 factors in studies of Western populations: cardiac causes (t = 6.32, P = 0.000) and smoking (t = 6.21, P = 0.000). This analysis revealed that publication bias affected 4 factors in the studies of Asian populations: BMI = 22.0-24.9 kg/m^2^ (t = -9.52, P = 0.000), BMI ≥25.0 kg/m^2^ (t = 3.36, P = 0.012), diabetes (t = 4.50, P = 0.003), smoking (t = 11.39, P = 0.000) and alcohol (t = 1.06, P = 0.008).

**Table 4:** **Egger's test & Sensitivity analysis**

Risk factors	Egger's test of Western(t value)	Egger's test of Western(P)	Sensitivity analysis of Western (HR 95% CI) ^◎^	Sensitivity analysis of Western(HR 95% CI) ^◇^	Egger's test of Asian ( ***t*** value)	Egger's test of Asian (P)	Sensitivity analysis of Asian (HR 95% CI) ^◎^	Sensitivity analysis of Asian (HR 95% CI) ^◇^
**BMI (kg/ m** ^**2**^ **)**								
22.0-24.9	1	N/A	1	1	1	N/A	1	1
18. 5–21. 9	-1.65	0.240	0.96 (0.93 ~ 0.99)^(1)^	0.97 (0.86 ~ 1.09)^(1)^	-9.52	0.000^*^	0.93 (0.90 ~ 0.97)^(9)^	0.97 (0.88 ~ 1.08)^(9)^
≥25.0	-0.48	0.652	1.02 (0.99 ~ 1.05)^(1)^	1.21 (0.99 ~ 1.48)^(1)^	3.36	0.012^*^	1.26 (1.22 ~ 1.31)^(9)^	1.28 (1.15 ~ 1.43)^(9)^
**SBP ( mm H g)**								
<120			1	1	1	N/A	1	1
120-139	N/A^▲^	N/A^▲^	1.28 (0.96 ~ 1.70)^(2)^	1.28 (0.95 ~ 1.73)^(2)^	N/A^▲^	N/A^▲^	1.96 (1.42 ~ 2.69)^(10)^	2.30 (1.03 ~ 5.09)^(10)^
≥140	N/A^▲^	N/A^▲^	2.25 (1.74 ~ 2.92)^(2)^	2.39 (1.44 ~ 3.97)^(2)^	N/A^▲^	N/A^▲^	4.69 (3.37 ~ 6.52)^(10)^	6.22 (2.27 ~ 17.08)^(10)^
**Hypertension**								
No	1	N/A	1	1	1	N/A	1	1
Yes	1.78	0.173	1.65 (1.43 ~ 1.90)^(3)^	1.79 (1.39 ~ 2.30)^(3)^	2.01	0.101	2.66 (2.29 ~ 3.10)^(11)^	2.84 (2.10 ~ 3.82)^(11)^
**Diabetes**								
No	1	N/A	1	1	1	N/A	1	1
Yes	1.17	0.280	1.72 (1.52 ~ 1.94)^(4)^	1.85 (1.43 ~ 2.38)^(4)^	4.50	0.003^*^	2.19 (2.01 ~ 2.39)^(12)^	1.95 (1.42 ~ 2.67)^(12)^
**Cardiac causes** ^**#**^								
No	1	N/A	1	1	1	N/A	1	1
Yes	6.32	0.000^*^	1.62 (1.50 ~ 1.75)^(5)^	1.74 (1.47 ~ 2.04)^(5)^	N/A^▲^	N/A^▲^	1.87 (1.45 ~ 2.42)^(13)^	1.86 (1.36 ~ 2.55)^(13)^
**Cardiac causes** (Atrial fibrillation)								
No	1	N/A	1	1	1^▲^	N/A	1	1
Yes	6.02	0.105	1.59 (1.33 ~ 1.90)^(6)^	1.59 (1.33 ~ 1.90)^(6)^	N/A^▲^	N/A^▲^	2.30 (1.50 ~ 3.53)^(14)^	2.30 (1.50 ~ 3.53)^(14)^
**Smoking**								
No	1	N/A	1	1	1	N/A	1	1
Yes	6.21	0.000^*^	1.97 (1.80 ~ 2.15)^(7)^	2.05 (1.68 ~ 2.49)^(7)^	11.39	0.000^*^	1.29 (1.12 ~ 1.48)^(15)^	1.27 (1.04 ~ 1.55)^(15)^
**Smoking** (Former)	2.09	0.171	1.20 (1.00 ~ 1.43)^(7)^	1.22 (0.94 ~ 1.59)^(7)^	N/A	N/A	N/A	N/A
**Smoking** (Current)	2.41	0.053	2.22 (1.96 ~ 2.53)^(7)^	2.27 (1.76 ~ 2.93)^(7)^	N/A	N/A	N/A	N/A
**Smoking** (≤20 Cigarettes/d)	-0.74	0.536	2.31 (1.80 ~ 2.96)^(7)^	2.31 (1.80 ~ 2.96)^(7)^	N/A	N/A	N/A	N/A
**Smoking** (>20 Cigarettes/d)	0.73	0.600	2.99 (2.31 ~ 3.86)^(7)^	2.97 (2.16 ~ 4.09)^(7)^	N/A	N/A	N/A	N/A
**Alcohol**								
No	1	N/A	1	1^▲^	1	N/A	1	1
Yes	-1.42	0.16	0.89 (0.76 ~ 1.04)^(8)^	0.92 (0.74 ~ 1.14)^(8)^	1.06	0.008^*^	1.28 (1.07 ~ 1.53)^(16)^	1.29 (1.07 ~ 1.57)^(16)^

*P values for publication bias; N/A, no data available; ^▲^Synthesis of References less than 3; BMI, Body mass index; ^#^Cardiac causes: Coronary heart disease, Left atrial hypertrophy, Angina, Atrial fibrillation; ◎Fixed effects model; ◇Random effects model; (1)-(16): (Additional files 1, 2, 3, 4, 5, 6, 7, 8, 9, 10, 11, 12, 13, 14, 15, 16).

By model transformation between the fixed effects model and random effects model for the sensitivity analyses of the included factors (Table 4). The sensitivity analysis results were consistent (Additional files 1, 2, 3, 4, 5, 6, 7, 8, 9, 10, 11, 12, 13, 14, 15, 16).

## Discussion

This systematic review of prospective cohort studies on the incidence of stroke summarized 7 risk factors for stroke that have been reported with relatively high frequency in prior studies. The prospective cohort studies incorporated into this review examined 867,782 Western or Asian participants and included investigations of large cohorts and studies of small community groups. To avoid biases attributable to differences in ethnicity and geography, the statistical analyses of the final combined results were stratified by Western or Asian population types.

Six factors affected the incidence of stroke in both Western and Asian populations. These factors included hypertension and diabetes, which are well-known risk factors for the incidence of stroke; thus, these findings are consistent with the results of earlier reports [66].

**Figure 2:**
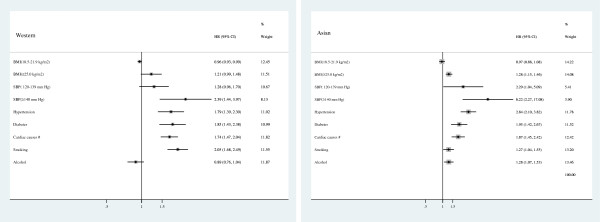
**Risk factors in Western and Asian populations.** (**Former, Current, ≤20 Cigarettes/d and >20 Cigarettes/d; #Cardiac causes: Coronary heart disease, Left Atrial hypertrophy, Angina, Atrial fibrillation; HR Hazard risk; CI, Confidence interval; BMI, Body mass index; SBP, Systolic blood pressure).

Studies have reported that a BMI ≥25 kg/m^2^ is associated with increased mortality among middle-aged populations, particularly from cardiovascular and cerebrovascular diseases, which are the leading causes of death [67]. Previous studies have reported similar findings for obesity [68]; in our study, obesity was one of the risk factors for the onset of disease in Asian populations, and an elevated BMI was associated with greater risk (Figure 2). In addition, the result of the risks associated with a BMI ≥25 kg/m^2^ was greater among Asian populations than among Western populations.

Stroke-related studies in the community of Framingham in the United States and the North Karelia region of Finland, as well as investigations of interventions for stroke-related risk factors in 7 Chinese cities, have confirmed the existence of a correlation between blood pressure and stroke [69–74]. SBP, a direct indicator of hypertension, is also a factor that directly influences the incidence of stroke. However, the risk of stroke was elevated in hypertensive patients relative to patients exhibiting high blood pressure without previously diagnosed hypertension [75]. The results of this study indicate that an increasing SBP is associated with an increased risk of stroke. In fact, the risk of stroke was greater among the participants with SBP ≥140 mm Hg than among the patients with diagnosed hypertension in both Asian and Western populations (Figure 2). A possible reason for this phenomenon is that most individuals diagnosed with hypertension have received appropriate anti-hypertensive interventions. Previous studies have shown that patients diagnosed with stroke often receive antihypertensive medication [15, 16]. From the results of our investigation, the risks associated with SBP ≥140 mm Hg is greater among Asian populations than among Western populations. For example, the cohort studies of a workforce in China [42],which examined steelworkers in Beijing, China, found an age-adjusted RR of 5.6 among these individuals, which was higher than the RR of 3.5 obtained in the Framingham study [76]. In our previous retrospective study [77], the Rothman and Keller [78] model was utilized to establish a multivariate model for predicting the incidence of stroke in human populations. Consistent with previous reports, this model represented the occurrence of stroke resulting from the combined long-term effects of multiple risk factors [79]. Our prior results also explored the effects of the interactions between hypertension and other risk factors on the incidence of stroke. However, the question of whether changes in other risk factors could alter the intensity of the risk of hypertension with respect to the incidence of stroke in a particular population (Western or Asian) merits additional study and discussion.

The Busselton Study reported that atrial fibrillation is a risk factor for stroke, with an RR of 5.9 [80], and other studies have also consistently indicated that atrial fibrillation is a risk factor for stroke [81]. In our investigation, atrial fibrillation was again identified as a risk factor; the result for this factor in Western populations was 2.30 (1.50 ~ 3.53), and there was no difference in Asian populations by interval estimation (Table 2). Consistent with our results, cardiovascular-related diseases (such as angina and CHD) were identified as risk factors for stroke as early as the Framingham Study and related reports [82]. Previous studies have suggested that left ventricular hypertrophy (LVH) is not correlated with the occurrence of stroke [13]; however, our investigation indicated that LVH is a risk factor for stroke in Western populations. Therefore, effective measures for preventing stroke in Western populations include lifestyle improvements, appropriate treatments, and reductions in the morbidities associated with chronic cardiovascular and cerebrovascular diseases.

Smoking is a risk factor for stroke, and the HR values for smoking-related behaviors were relatively high in Western populations. After stratifying the results based on the periods during which smoking occurred, it was determined that former smoking had no effect on the incidence of stroke in Western populations. Certain prior studies have suggested that being a former smoker can affect the incidence of stroke [83–85], whereas other studies have found no significant effects associated with this characteristic [86]. We observed a dose–response association for the number of cigarettes smoked per day in Western populations: the HR value associated with smoking more than 20 cigarettes per day was much greater than the HR values of other risk factors by point estimation (Figure 2); this result is consistent with the findings of previous studies [86, 87]. Therefore, reducing daily cigarette consumption is an effective preventive measure for reducing the incidence of stroke.

The results from the studies of Asian populations indicated that long-term alcohol consumption was also a risk factor for stroke, although this factor had no effect on the incidence of stroke in Western populations (Table 3). Prior studies have also produced controversial results with respect to the significance of this factor: certain studies have determined that heavy long-term alcohol consumption is a risk factor for stroke [88], but other studies have reached the opposite conclusion [89]. However, heavy long-term alcohol consumption is a risk factor for many chronic diseases, and therefore, limiting alcohol consumption may play an indirect role in preventing the incidence of stroke.

### Limitations

This systematic review and meta-analysis used the Newcastle-Ottawa scale to score all of the included studies. Overall, the quality of the studies on Chinese populations was significantly lower than the quality of the studies on other populations (T-Test: Z = 3.015, P = 0.007). The primary reason for this finding was that the populations examined in the former studies were mostly large, long-term, community-based cohorts. For example, the studies by the British regional heart study [24] and the PROCAM study [29, 30], among others, were based on data from large European cohorts [29, 30, 32]. By contrast, most of the included Asian studies examined small community-based cohorts or even hospital cases. For example, the research participants assessed by the cadre's sanitarium of Xi’an, China [37] were recruited from a sanatorium for retired military cadres in Xi'an, China. Result biases may be relatively low for large community cohorts but higher for smaller samples. In addition, bias may also be related to the dropout rate. For instance, the study in Shanghai, China [53] reported a dropout rate of 3.09%, whereas the CMCS study [44] reported a dropout rate of up to 22.5%. Although this investigation involved the examination of a large number of literature databases, there was less investigation of the unpublished literature and conference-related information, potentially creating publication bias.

Furthermore, only a limited number of studies were included in this investigation; thus, a large amount of data regarding risk factors was missing. In addition, few studies were pooled to obtain certain data points, particularly for Asian populations, and the number of studies finally included in the meta-analysis was very low for individual risk factors (usually in the range of 1 to 10), and this issue might have produced bias in the results. Therefore, these limitations are related to the absence of studies from South Asia and West Asia, and we believe this might be one reason for the publication bias. Consequently, to obtain more reliable data and results, additional large-sample, multi-center, randomized controlled studies and/or long-term cohort studies must be conducted.

## Conclusions

This meta-analysis presents the data from large-scale prospective cohort studies to summarize and evaluate the risk factors for stroke in Western and Asian populations. The results of this investigation are credible, and the pooled results obtained in this review are more reliable than the findings from prior small-scale cohort studies and case–control studies. The analyses conducted in this investigation revealed that Western and Asian populations differ with respect to the factors that affect the incidence of stroke. Therefore, different interventional approaches should be implemented to reduce the risk of stroke among high-risk individuals from different regions and different ethnic groups.

## Electronic supplementary material

Additional file 1: **BMI of Western, Body Mass Index** **(1. 25.0-25.9 Kg/m** ^**2**^ **, 2. ≥26.0 Kg/m** ^**2**^ **; Left: Fixed effects model, Right: Random effects model).** (DOC 38 KB)

Additional file 2: **SBP of Western, Systolic blood pressure (1. 120–129 mm Hg, 2. 130–139 mm Hg, 3. 140-149 mm Hg2. 150-159 mm Hg2. ≥160 mm Hg; Left: Fixed effects model, Right: Random effects model).** (DOC 32 KB)

Additional file 3: **Hypertension of Western (Left: Fixed effects model, Right: Random effects model).** (DOC 29 KB)

Additional file 4: **Diabetes of Western (Left: Fixed effects model, Right: Random effects model).** (DOC 30 KB)

Additional file 5: **Cardiac causes of Western (1. Angina, 2. Coronary Heart Disease, 3. Atrial fibrillation, 4. Left ventricular hypertrophy; Left: Fixed effects model, Right: Random effects model).** (DOC 28 KB)

Additional file 6: **Cardiac causes** **-Atrial** **fibrillation of Western (Left: Fixed effects model, Right: Random effects model).** (DOC 28 KB)

Additional file 7: **Smoking of Western (1. < 20 Cigarettes/d, 2. =20 Cigarettes/d, 3. > 20 Cigarettes/d; Left: Fixed effects model, Right: Random effects model).** (DOC 48 KB)

Additional file 8: **BMI of Asian, Body Mass Index (1. 25.0-25.9 Kg/m** ^**2**^ **, 2. ≥26.0 Kg/m** ^**2**^ **; Left: Fixed effects model, Right: Random effects model).** (DOC 46 KB)

Additional file 9: **SBP of Asian, Systolic blood pressure (1. 120–129 mm Hg, 2. 130–139 mm Hg, 3. 140-149 mm Hg2. 150-159 mm Hg2. ≥160 mm Hg; Left: Fixed effects model, Right: Random effects model).** (DOC 34 KB)

Additional file 10: **Hypertension of Asian (Left: Fixed effects model, Right: Random effects model).** (DOC 32 KB)

Additional file 11: **Diabetes of Asian (Left: Fixed effects model, Right: Random effects model).** (DOC 34 KB)

Additional file 12: **Cardiac causes of Asian (1. Angina, 2. Coronary Heart Disease, 3. Atrial fibrillation, 4. Left ventricular hypertrophy; Left: Fixed effects model, Right: Random effects model).** (DOC 31 KB)

Additional file 13: **Cardiac causes -Atrial fibrillation of Asian (Left: Fixed effects model, Right: Random effects model).** (DOC 30 KB)

Additional file 14: **Smoking of Asian (Fixed effects model, Right: Random effects model).** (DOC 33 KB)

Additional file 15: Alcohol of Asian (*Ischemic stroke, #Hemorrhagic stroke; Left: Fixed effects model, Right: Random effects model). (DOC 32 KB)

Additional file 16: Alcohol of Western (*Ischemic stroke, #Hemorrhagic stroke; Left: Fixed effects model, Right: Random effects model). (DOC 33 KB)

## Abbreviations

HR: Hazard ratio
CI: Confidence interval
BMI: Body mass index
SBP: Systolic blood pressure
MOOSE: Meta-analysis of observational studies in epidemiology
CBM: Chinese biomedical literature database
CMCI/CMCC: Chinese medical citation index/chinese medical current contents
CENTRAL: Cochrane central register of controlled trials
RR: Relative risk
NOS: Newcastle-ottawa scale
CHD: Coronary heart disease
LVH: Left ventricular hypertrophy.
