# Supplementary material for: Risk Factors of Stroke in Western and Asian Countries: A Systematic Review and Meta-analysis of Prospective Cohort Studies
Source: BMC Public Health. 2014 Jul 31;14:776. doi: 10.1186/1471-2458-14-776 (PMC4246444; doi:10.1186/1471-2458-14-776)
Supplement: Supplementary file 4 — Additional file 4: Diabetes of Western (Left: Fixed effects model, Right: Random effects model). (DOC 30 KB) [file 12889_2014_7280_MOESM4_ESM.doc]

Additional file 4.
