# Supplementary material for: Risk Factors of Stroke in Western and Asian Countries: A Systematic Review and Meta-analysis of Prospective Cohort Studies
Source: BMC Public Health. 2014 Jul 31;14:776. doi: 10.1186/1471-2458-14-776 (PMC4246444; doi:10.1186/1471-2458-14-776)
Supplement: Supplementary file 7 — Additional file 7: Smoking of Western (1. < 20 Cigarettes/d, 2. =20 Cigarettes/d, 3. > 20 Cigarettes/d; Left: Fixed effects model, Right: Random effects model). (DOC 48 KB) [file 12889_2014_7280_MOESM7_ESM.doc]

Additional file 7.
