# Supplementary material for: Risk Factors of Stroke in Western and Asian Countries: A Systematic Review and Meta-analysis of Prospective Cohort Studies
Source: BMC Public Health. 2014 Jul 31;14:776. doi: 10.1186/1471-2458-14-776 (PMC4246444; doi:10.1186/1471-2458-14-776)
Supplement: Supplementary file 8 — Additional file 8: BMI of Asian, Body Mass Index (1. 25.0-25.9 Kg/m 2 , 2. ≥26.0 Kg/m 2 ; Left: Fixed effects model, Right: Random effects model). (DOC 46 KB) [file 12889_2014_7280_MOESM8_ESM.doc]

Additional file 8.
