# Supplementary material for: Risk Factors of Stroke in Western and Asian Countries: A Systematic Review and Meta-analysis of Prospective Cohort Studies
Source: BMC Public Health. 2014 Jul 31;14:776. doi: 10.1186/1471-2458-14-776 (PMC4246444; doi:10.1186/1471-2458-14-776)
Supplement: Supplementary file 15 — Additional file 15: Alcohol of Asian (*Ischemic stroke, #Hemorrhagic stroke; Left: Fixed effects model, Right: Random effects model). (DOC 32 KB) [file 12889_2014_7280_MOESM15_ESM.doc]

Additional file 15.
